# Evaluation of the Accuracy and Performance of Two Commercial Pregnancy‐Associated Glycoprotein Tests for Early Pregnancy Detection in Cows

**DOI:** 10.1002/vms3.70226

**Published:** 2025-01-28

**Authors:** Tarik Safak, Kenan Çağrı Tümer, Yağmur İpek Alp, Taha Yasin Özen, Oznur Yilmaz‐Koc

**Affiliations:** ^1^ Department of Obstetrics and Gynecology Faculty of Veterinary Medicine Kastamonu University Kastamonu Turkey; ^2^ Department of Internal Medicine Faculty of Veterinary Medicine Kastamonu University Kastamonu Turkey; ^3^ Sanfa Agriculture and Livestock Kastamonu Turkey; ^4^ Department of Obstetrics and Gynecology Faculty of Veterinary Medicine Siirt University Siirt Turkey

**Keywords:** cow, Milk Pregnancy Test, OnFarm Pregnancy Test, pregnancy‐associated glycoproteins

## Abstract

The aim of this study was to determine and compare the diagnostic accuracies of two commercial pregnancy‐associated glycoprotein tests, Alertys OnFarm Pregnancy Test (AOPT) and Alertys Milk Pregnancy Test (AMPT), for early pregnancy diagnosis in dairy cattle. Holstein cows (*n* = 124) were used in the study. Whole blood samples were collected from the jugular vein 28 days after fixed‐time artificial insemination (FTAI). In addition, teats of these cows were swabbed and milk samples were collected into sterile Falcon tubes. AOPT was performed on the farm within 2 h after whole blood collection. Milk samples for AMPT analysis were taken to the laboratory and analysed within 2 h. Transrectal ultrasonography was performed on the 32nd day after FTAI as a reference test. Comparative evaluation was made according to the AOPT and AMPT results, 28 days after FTAI. The sensitivity, specificity, accuracy, positive predictive value (PPV) and negative predictive value (NPV) for the AOPT were 92.4%, 80.0%, 87.9%, 89.0% and 85.7% and for AMPT were 97.5%, 82.2%, 91.9%, 90.6% and 94.9% respectively. Cohen's kappa statistic showed a 91.9% agreement (kappa = 0.820, *p* < 0.001) between the reference test and AMPT, and an 87.9% agreement (kappa = 0.735, *p* < 0.001) between the reference test and AOPT. AOPT and AMPT offers a reliable, non‐invasive (in milk) and practical approach to pregnancy diagnosis in cows. These methods enable early pregnancy detection and can be easily integrated into farm routines, enhancing reproductive management and overall herd productivity.

## Graphical abstract

- Early pregnancy diagnosis contributes to increasing productivity and profitability in dairy farms.
- The performance of AOPT and AMPT used for early pregnancy diagnosis in dairy cattle was evaluated for the first time together.
- AOPT and AMPT are practical methods for early pregnancy diagnosis, 28 days after insemination in dairy cows.

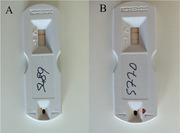

## 1 Introduction

In dairy herd management, maintaining reproductive efficiency requires the early detection of pregnancy following artificial insemination. Cows that are not pregnant should be identified quickly and reinseminated without delay. Pregnant and non‐pregnant cows have to be diagnosed as quickly as possible. Therefore, early pregnancy diagnosis in dairy farms is crucial for economic profitability (Inchaisri et al. 2010; Ott et al. 2014; Whitlock and Maxwell 2008).

Over the decades, various direct and indirect methods have been developed for early pregnancy diagnosis in cattle. However, for these methods to be widely adopted in practice, they must be easy to apply, cost‐effective and reliable. Amongst the direct methods, rectal palpation and transrectal ultrasonography are most commonly used. Rectal palpation is simple, fast, easy to perform and inexpensive, but a reliable pregnancy diagnosis can only be made after the 35th day post‐insemination. Moreover, there is a risk of pregnancy loss due to manipulations used to detect uterine asymmetry, fluctuation, amniotic sac and the conceptus (Balhara et al. 2013; Peter 2013; Romano et al. 2007). In contrast, transrectal ultrasonography allows for earlier pregnancy detection (5–7 days) than rectal palpation, which enables monitoring of the embryo's viability by detecting its heartbeat, and poses a lower risk of iatrogenic pregnancy loss (Romano et al. 2006).

Indirect methods have been developed as alternatives to direct approaches for pregnancy diagnosis in cattle. These methods involve the qualitative or quantitative detection of various pregnancy‐related substances in maternal body fluids like blood and milk. Molecules such as pregnancy‐specific protein B (PSPB), early pregnancy factor (EPF), estrone sulphate, progesterone (P4), interferon tau (IFN‐τ) and pregnancy‐associated glycoproteins (PAGs) are commonly investigated (Ott et al. 2014; Ott and Gifford 2010). PAGs are part of a large family of inactive aspartic proteinases expressed by the placenta in ruminants, including cattle, sheep and goats (Haugejorden et al. 2006). PSPB was the first pregnancy‐specific marker identified in cattle, produced by the placenta (Butler et al. 1982) and later reclassified as bovine PAG‐1 (Green et al. 2005). To date, at least 22 bovine PAG genes, detectable in maternal blood, have been identified. From the 25th day of pregnancy, PAGs enter the maternal bloodstream (Barbato et al. 2022; Whitlock and Maxwell 2008).

A significantly recent advancement is the development of the enzyme‐linked immunosorbent assay (ELISA), which is used to detect circulating PAGs in maternal blood for pregnancy diagnosis (Green et al. 2005). It has been reported that serum PAG concentrations can be detected 15–35 days post‐insemination in cows (Karakuş et al. 2021; Oliveira Filho et al. 2020; Lobago et al. 2009; Zoli et al. 1992), 19–24 days in buffaloes (Karen et al. 2007) and 28 days in sheep (Yotov and Sinapov 2023) and goats (Akkaya Doğan and Köse 2022). Thus, it has been reported that the ELISA method has become useful in early pregnancy diagnosis. Commercial test kits are currently available for detecting PAG in plasma, serum and whole blood in dairy cattle (Lopez‐Gatius et al. 2024). While a number of kits are still under development and field studies for several have been completed (Mayo et al. 2016), others still require testing and validation to verify their reliability and accuracy. Recently, new commercial pregnancy test kits have been introduced for detecting PAG in milk, plasma, serum and whole blood samples. For instance, the lateral flow test and Alertys OnFarm Pregnancy Test (AOPT) have been used to diagnose pregnancy in cows in whole blood (Akköse 2023). In addition, the Alertys Milk Pregnancy Test (AMPT) was developed to detect PAG in milk and validated in buffaloes (Arshad et al. 2022). However, AMPT for pregnancy diagnosis in cattle has not yet been assessed and necessitates more validation studies for application in cattle.

This study aims to evaluate and validate the diagnostic performance of AOPT in whole blood and AMPT in milk on the 28th day after fixed‐time artificial insemination (FTAI), using transrectal ultrasonography on the 32nd day after FTAI in Holstein cows as the reference standard.

## 2 Materials and Methods

### 2.1 Ethical Statement

This study was approved by the Kastamonu University Animal Experiments Local Ethics Committee (date: 21 September 2023, approval number: 2023/35).

### 2.2 Animals and Synchronisation Protocol

**FIGURE 1:**
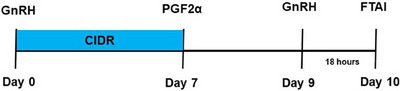
Hormonal protocol used in cows (CIDR‐Ovsynch). CIDR, controlled internal drug release; FTAI, fixed‐time artificial insemination; GnRH, gonadotropin‐releasing hormone; PGF_2_α, prostaglandin F_2_α.

The study was conducted at a private dairy farm in the Kastamonu province of Türkiye, which has a total herd capacity of 2000 Holstein dairy cattle. In the study, 4–5‐year‐old multiparous (second and third lactation, days in milk 65–90 days) Holstein cows (*n* = 124) were used. The CIDR‐Ovsynch protocol was applied to all cows, as detailed depicted in Figure 1. Briefly, on Day 0, a Controlled Internal Drug Release (CIDR, Zoetis, Türkiye) device containing 1.38 g of P4 was inserted intra‐vaginally, and 100 µg of the gonadotropin‐releasing hormone (GnRH) analogue gonadorelin (Ovarelin, Ceva, Türkiye) was administered intra‐muscularly. On the seventh day, 25 mg of prostaglandin F_2_α (PGF2α) analogue dinoprost (Enzaprost, Ceva, Türkiye) was administered simultaneously with the removal of the CIDR device, followed by a second GnRH analogue injection on Day 9. FTAI was performed 18 h after the second GnRH injection (Meglić et al. 2022; Seker et al. 2017).

### 2.3 Pregnancy Diagnosis From Whole Blood Sample

**FIGURE 2:**
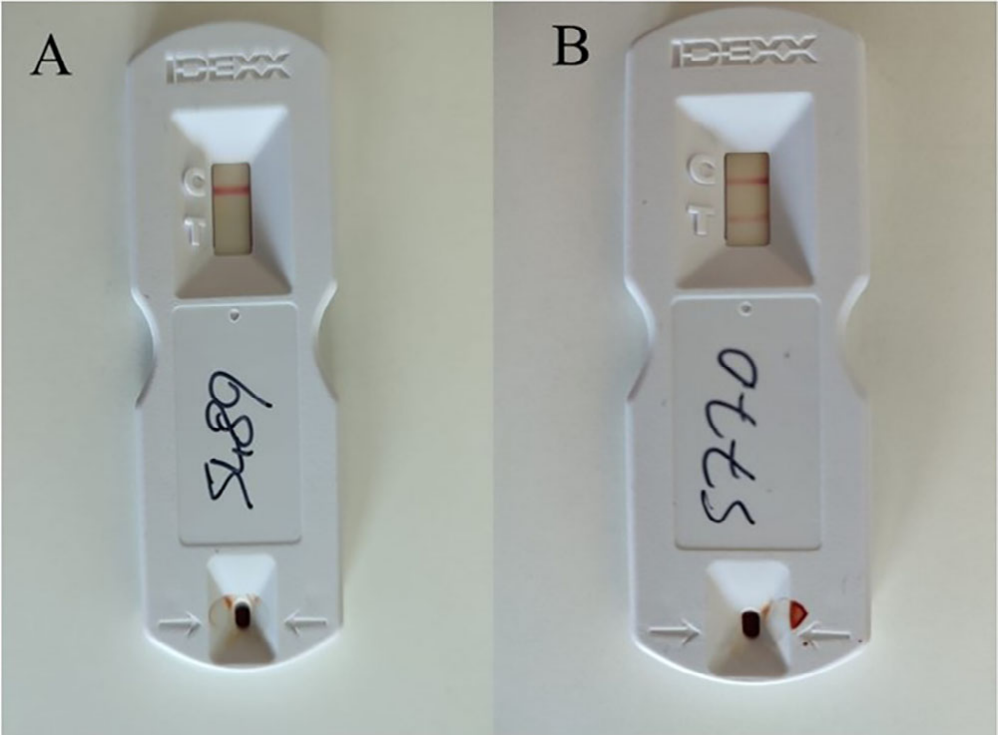
Alertys OnFarm Pregnancy Test of two cows on the farm. The test (T) line may vary from very light to dark. The presence of any intensity of colour on the T line is considered Pregnant. The absence of the T line indicates that the animal is non‐pregnant. (A) Non‐pregnant and (B) pregnant.

A commercial AOPT kit (Idexx Laboratories, Westbrook, ME, USA) was utilised to detect pregnancy in whole blood following the manufacturer's instructions and as outlined in the literature (Akköse 2023). A blood sample was collected from the jugular vein of cows into tubes containing tripotassium ethylenediaminetetraacetic acid (K3‐EDTA) (BD Vacutainer, Plymouth, UK) on the 28th day post‐FTAI. Four drops of whole blood and six drops of washing solution were added to the test well of the kit, followed by a 20‐min incubation. After incubation, the appearance of two lines (C and T) indicated pregnancy, while a single line (C) signified non‐pregnancy (Figure 2).

### 2.4 Pregnancy Diagnosis by Milk Sample

**FIGURE 3:**
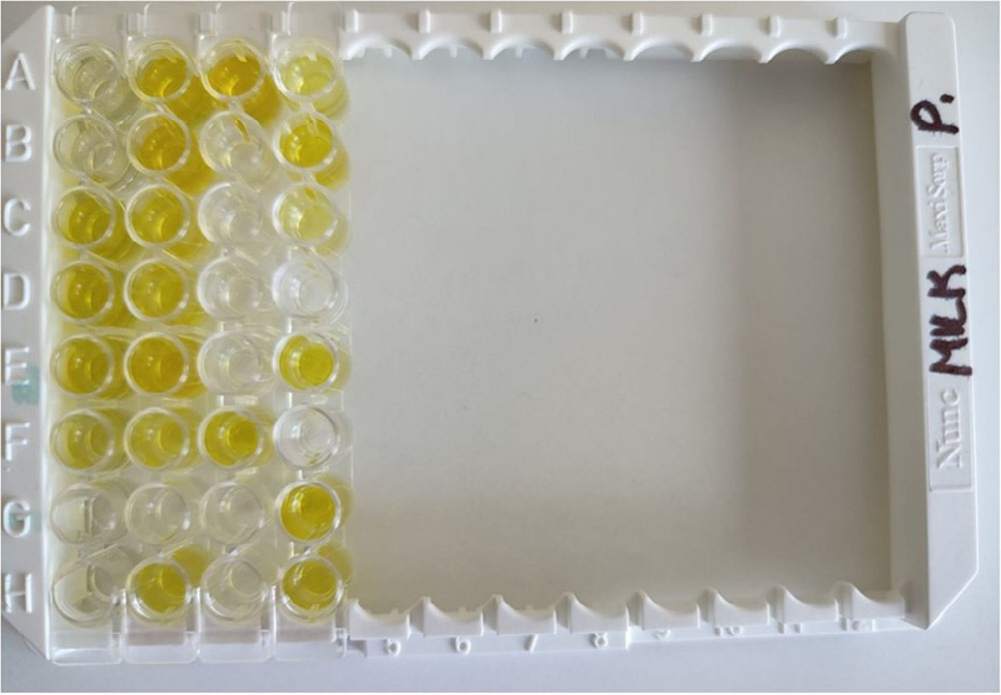
Image of the Alertys Milk Pregnancy Test of 28 cows on the farm. 1H and 3H wells: Negative control, 2H and 4H wells: Positive control. Other wells on the plate belong to milk samples. There has been a yellow colour change with S‐N value ≥ 0.25. Wells without yellow colour change have S‐N value < 0.25. Yellow colour: Pregnant, without yellow colour: Non‐pregnant.

Milk samples were collected by thoroughly cleaning all four teats with water and a disinfectant solution (povidone iodine 10%, Povilon, Medisin, Türkiye). The first few squirts of milk were discarded, and 5 mL of milk was then collected from each cow into 15 mL Falcon tubes under sterile conditions on the 28th day after FTAI. PAG concentrations were measured in the collected milk samples using the AMPT kit (Idexx Laboratories, Westbrook, ME, USA), following the procedures as described in the literature (Arshad et al. 2022). The analysis method was as follows: 150 µL of negative and positive controls were added to two separate wells of the plate, followed by the addition of 150 µL of milk sample to the appropriate wells. The wells were sealed and incubated at 37°C for 120 min. After incubation, optical density (OD) values were calculated according to the manufacturer's instructions and was read by using a microtiter plate reader at 450 nm wavelength. Results were calculated from the OD of the sample [the reference wavelength OD of the sample (S) − the OD of the negative control (N) at 450 nm (with both values corrected by subtraction of the reference wavelength OD of the negative control)], which resulted in an S‐N value. In interpreting the milk PAG OD levels, cows were classified as pregnant if the S‐N value was ≥ 0.25 and non‐pregnant if the S‐N value was < 0.25 (Arshad et al. 2022) (Figure 3).

### 2.5 Pregnancy Diagnosis Using Transrectal Ultrasonography

Diagnosis of pregnancy was performed by the same veterinarian using transrectal ultrasonography on Day 32 after FTAI. A real‐time B‐mode ultrasound device (Hasvet 838, Hasvet, Türkiye) equipped with a portable 7.5 MHz linear probe was used for the transrectal examination (Risvanli et al. 2022). A positive pregnancy diagnosis was based on the observation of a fluid‐filled uterus containing an embryo with a detectable heartbeat. If a uterus without an embryo was observed, the pregnancy was considered negative. In addition, the presence of a fluid‐filled uterus containing an embryo without a heartbeat or embryonic membrane integrity damage, was considered evidence of embryonic death, and these cases were included in the pregnancy‐negative group.

### 2.6 Statistical Analysis

Pregnancy results from transrectal ultrasonography, used as the reference test, were compared with the results of PAG tests in whole blood and milk samples. Sensitivity, specificity, positive predictive value (PPV), negative predictive value (NPV) and accuracy were calculated for both test methods. For each test, the true positive (Tp; ultrasound result pregnant, test result pregnant), false positive (Fp; ultrasound result non‐pregnant, test result pregnant), false negative (Fn; ultrasound result pregnant, test result non‐pregnant) and true negative (Tn; ultrasound result non‐pregnant, test result non‐pregnant) results were identified. Using these data, sensitivity [Tp/(Tp + Fn) × 100], specificity [Tn/(Tn + Fp) × 100], PPV [Tp/(Tp + Fp) × 100], and NPV [Tn/(Tn + Fn) × 100] were calculated for both methods. The effectiveness of the tests in pregnancy diagnosis was compared by calculating the accuracy rates [(Tp + Tn)/(Tp + Fp + Fn + Tn) × 100] (Kaya et al. 2017).

The area under the curve (AUC) values obtained from receiver operator characteristic (ROC) curve analysis for AMPT and AOPT were compared using DeLong's test in RStudio, with the ‘pROC’ package (Robin et al. 2011). In addition, agreement between the reference method and the test methods was assessed using the kappa test in RStudio, utilising the ‘irr’ package. Kappa test results were interpreted as follows: 0.00–0.20, slight agreement; 0.21–0.40, fair agreement; 0.41–0.60, moderate agreement; 0.61–0.80, substantial agreement and 0.81–1.00, perfect agreement. A *p* value below 0.05 was considered statistically significant (McHugh 2012).

## 3 Results

Based on transrectal ultrasonography findings on Day 32 after FTAI, 79 cows (63.71%) were diagnosed as pregnant and 45 cows (36.29%) were non‐pregnant. The analysis with AOPT revealed that out of 82 cows identified as pregnant by the test, 73 were Tp and 9 were Fp. In addition, out of 42 cows identified as non‐pregnant, 36 were Tn and 6 were Fn (Table 1).

**TABLE 1:** Evaluation of the diagnostic test characteristics for determining the pregnancy status 32 days after fixed‐time artificial insemination, considering transrectal ultrasonography as the gold standard.

		Transrectal ultrasonography		
		Pregnant	Non‐pregnant	Total
AOPT	Pregnant	73 (Tp)	9 (Fp)	82
	Non‐pregnant	6 (Fn)	36 (Tn)	42
	Total	79	45	124

Abbreviations: AOPT: Alertys OnFarm Pregnancy Test, Fn: false negative, Fp: false positive, Tn: true negative, Tp: true positive.

In the AMPT analysis, 85 cows were detected as pregnant. Of these, 77 were Tp and 8 were Fp. Amongst the 39 non‐pregnant cows, 37 were Tn and 2 were Fn (Table 2).

**TABLE 2:** Evaluation of the diagnostic test characteristics of the AMPT for determining the pregnancy status 32 days after fixed‐time artificial insemination, considering transrectal ultrasonography as the gold standard.

		Transrectal ultrasonography		
		Pregnant	Non‐pregnant	Total
AMPT	Pregnant	77 (Tp)	8 (Fp)	85
	Non‐pregnant	2 (Fn)	37 (Tn)	39
	Total	79	45	124

Abbreviations: AMPT, Alertys Milk Pregnancy Test; Fn, false negative; Fp, false positive; Tn, true negative; Tp, true positive.

The sensitivity, specificity, accuracy, PPV and NPV for AOPT and AMPT are summarised in Table 3. For AOPT, the sensitivity was 92.4%, specificity was 80.0%, accuracy was 87.9%, PPV was 89.0% and NPV was 85.7%. In comparison, AMPT exhibited a sensitivity of 97.5%, specificity of 82.2%, accuracy of 91.9%, PPV of 90.6% and NPV of 94.9%.

**TABLE 3:** Sensitivity, specificity, accuracy, positive predictive values and negative predictive values of the AOPT and AMPT.

	Sensitivity (%) (CI_95_)	Specificity (%) (CI_95_)	Accuracy (%) (CI_95_)	PPV (%) (CI_95_)	NPV (%) (CI_95_)
AOPT	92.4 (84.2–97.2)	80.0 (65.4–90.4)	87.9 (80.8–93.1)	89.0 (80.2–94.9)	85.7 (71.5–94.6)
AMPT	97.5 (91.2–99.7)	82.2 (67.9–92.0)	91.9 (85.7–96.1)	90.6 (82.3–95.8)	94.9 (82.7–99.4)

Abbreviations: AMPT, Alertys Milk Pregnancy Test; AOPT, Alertys OnFarm Pregnancy Test; CI95, 95% confidence interval; NPV, negative predictive value; PPV, positive predictive value.

The AUC values for AMPT and AOPT were 0.927 and 0.873, respectively. DeLong's test indicated no significant difference between the AUC values of the two tests (*p* = 0.185). Cohen's kappa statistic showed a 91.9% agreement (kappa = 0.820, *p* < 0.001) between the reference test and AMPT, and an 87.9% agreement (kappa = 0.735, *p* < 0.001) between the reference test and AOPT.

## 4 Discussion

Ultrasound and rectal palpation are primary methods for detecting pregnancy in dairy cows. However, the accuracy of these methods largely depends on the operator's skill and experience. Inexperienced staff or incorrect pregnancy detection can lead to issues such as pregnancy loss in pregnant cows and an extended empty gestation period. This, in turn, can reduce the lifetime milk production of non‐pregnant cows (Yáñez et al. 2023). Various pregnancy markers have been studied for use in different livestock species, including sheep, (Ishwar 1995) goats (Karadaev 2015), buffaloes (Barbato and Barile 2012) and cows (Balhara et al. 2013). With advancements in time and scientific research, numerous companies are now successfully developing commercial assays for pregnancy diagnosis across various livestock species, utilising PAGs in serum, plasma and milk as the basis for these assays (Commun et al. 2016).

In this study, two different commercial PAG pregnancy tests were evaluated using both whole blood and milk samples. Consistent with our findings, Akköse (2023) reported the sensitivity, specificity, PPV, NPV and accuracy of the AOPT on Day 28 post‐AI as 100%, 93.1%, 89.1%, 100% and 95.6%, respectively. The AOPT showed 100% sensitivity and 81.6% specificity in heifers. For multiparous cows, this test had a sensitivity of 98.5% and a specificity of 89.5% between Days 28 and 34 of pregnancy. In addition, the accuracy, sensitivity, specificity, PPV and NPV of the lateral diffusion test were reported as 98.9%, 88.7%, 86.8% and 99.1%, respectively, in a comprehensive evaluation of both primiparous and multiparous Holstein Friesian dairy cattle (Szelényi et al. 2024). Sensitivity and specificity of pregnancy tests can vary based on factors such as parity and breed differences. It should also be observed that maternal PAG concentrations differ between heifers with high and low fertility levels (Reese et al. 2019).

Karakuş et al. (2021) achieved high accuracy in diagnosing pregnancy at 30 days post‐AI using the commercial visual test kit (Fassisi BoviPreg) in serum samples from Simmental and Brown Swiss cows. Sensitivity, specificity, accuracy, PPV and NPV for the Rapid Visual Pregnancy Test (RVPT) on Day 28 post‐AI in serum samples were reported as 98%, 85%, 92%, 87% and 98%, respectively (Mayo et al. 2016). In another study, Akköse (2023) found that the RVPT had sensitivity, specificity, accuracy, PPV and NPV values of 97.4%, 92.1%, 94%, 98.4% and 94%, respectively.

Ricci et al. (2015) reported that for Holstein cows 32 days post‐AI using the plasma PAG‐ELISA based kit (Idexx Bovine Pregnancy Test), the accuracy was 92%, with sensitivity and specificity of 100% and 87%, respectively. For goats, using the RVPT with ultrasonographic results as the gold standard, the sensitivity was 94.12%, while specificity, PPV, NPV and accuracy were 80.49%, 80%, 94.29% and 86.67%, respectively (Akkaya Doğan and Köse 2022). In sheep, the same test showed sensitivity, specificity, PPV, NPV and accuracy values of 95.79%, 52.38%, 90.10%, 73.33% and 87.93%, respectively (Akköse et al. 2022). The validity and reliability of the RVPT were consistent across studies in goats and sheep, showing similar effectiveness in detecting pregnancies as observed in cows. Using milk samples for pregnancy detection is a preferable approach as it minimises stress for lactating animals, which is crucial for maintaining their milk productivity (Yang et al. 2024). In our study, the AMPT demonstrated sensitivity of 97.5%, specificity of 82.2%, accuracy of 91.9%, PPV of 90.6% and NPV of 94.9%. In contrast, Arshad et al. (2022) reported that the same AMPT had 77.6% sensitivity, 89.1% specificity, 79% PPV, 88.3% NPV and 85.1% accuracy in Nili‐Ravi buffaloes at 24 days post‐AI. In addition, a study using a small sample size of Bulgarian water buffaloes (*n* = 22) found that a milk‐based PAGs‐ELISA test had 100% specificity and approximately 100% sensitivity at around 25 days post‐AI (Tadeo et al. 2021).

In the study by Durocher et al. (2022), a milk PAG‐ELISA test conducted between 23 and 27 days after AI achieved sensitivity and specificity values of 98% using a standard corrected OD threshold of 0.15 in Holstein cows. In contrast, our study utilised a threshold of 0.25 OD, resulting in sensitivity and specificity values of 97.5% and 82.2%, respectively. This indicates that while our study's sensitivity and specificity are slightly lower compared to the study performed by Durocher et al. (2022), differences in OD thresholds may contribute to these variations.

One of the reasons for Fp results may be early embryonic death. Previous studies have shown that the half‐life time of PAGs is approximately 8 days (Mialon et al. 1993). Therefore, although embryonic death has occurred, the decrease in PAG concentration in the maternal circulation may be delayed or high concentrations may still be present. This may cause Fp pregnancy test results (Bragança et al. 2018; Whitlock and Maxwell 2008). Another reason is that detectable PAG concentrations are still detected in the circulation during the post‐partum period (De Sousa et al. 2003; Haugejorden et al. 2006). Furthermore, the presence of inflammatory diseases such as endometritis, metritis, and mastitis during the post‐partum period slows the decrease in PAG concentration (Priyo et al. 2024). The RVPT and AOPT produced 16 and 14 Fp results, respectively. The same animal yielded 13 Fp results in both tests, whereas the AOPT and RVPT produced only 1 and 3 Fp results, respectively. In four of these cases, embryonic mortality was detected by ultrasonography (Akköse 2023). In our study, despite the absence of embryonic death or post‐partum diseases, we observed Fp results with the AOPT kit in nine cows and with the AMPT in eight cows. Priyo et al. (2024) and Haugejorden et al. (2006), stated that although PAG concentration decreased in the post‐partum process, it could still be detected. It is thought that the Fp results in our study may be due to the continued presence of PAG concentrations in the post‐partum period.

In conclusion, PAG analysis via AOPT (in whole blood) and AMPT (in milk) offers a practical and non‐invasive approach (in the case of milk) for pregnancy diagnosis in cows. Ideally, a diagnostic test should provide virtually error‐free Tn results to reliably identify non‐pregnant animals within the herd. However, the tests evaluated in this study demonstrated moderate specificity and relatively good accuracy. While these tests allow for early detection of pregnancy and are easily integrated into farm routines, their specificity and accuracy constraints warrant cautious interpretation of negative results. To optimise herd management and productivity, verifying negative test outcomes through ultrasound examination is strongly recommended.

## Author Contributions

**Tarik Safak**: conceptualisation, methodology, project administration, writing–original draft, writing–review and editing, supervision. **Kenan Çağrı Tümer**: investigation, writing–review and editing, formal analysis, writing–original draft. **Yağmur İpek Alp**: investigation, funding acquisition. **Taha Yasin Özen**: formal analysis. **Oznur Yilmaz‐Koc**: investigation, formal analysis.

## Ethics Statement

This study was approved by the Kastamonu University Animal Experiments Local Ethics Committee (date: 21 September 2023, approval number: 2023/35).

## Conflicts of Interest

The authors declare no conflicts of interest.

## Data Availability

The data that support the findings of this study are available from the corresponding author upon reasonable request.
